# Single injection of sustained-release prostacyclin analog ONO-1301-MS ameliorates hypoxic toxicity in the murine model of amyotrophic lateral sclerosis

**DOI:** 10.1038/s41598-019-41771-4

**Published:** 2019-03-27

**Authors:** Satoru Tada, Tatsusada Okuno, Mikito Shimizu, Yoshiki Sakai, Hisae Sumi-Akamaru, Makoto Kinoshita, Kazuya Yamashita, Eri Sanda, Chi-Jing Choong, Akiko Namba, Tsutomu Sasaki, Toru Koda, Kazushiro Takata, Shigeru Miyagawa, Yoshiki Sawa, Yuji Nakatsuji, Hideki Mochizuki

**Affiliations:** 1 Department of Neurology, Osaka University Graduate School of Medicine, 2-2, Yamadaoka, Suita, Osaka 565-0871 Japan; 2 Department of Neurology, Tanaka Naika Medical Clinic, 2-7-28, Matsunohama-cho, Izumiotsu, Osaka 595-0072 Japan; 3 Department of Cardiovascular Surgery, Osaka University Graduate School of Medicine, 2-2, Yamadaoka, Suita, Osaka 565-0871 Japan

## Abstract

Amyotrophic lateral sclerosis (ALS) is a progressive neurodegenerative disease characterized by several pathologies including oxidative stress, apoptosis, neuroinflammation, and glutamate toxicity. Although multiple reports suggest that ischemia and hypoxia in the spinal cord plays a pivotal role in the pathogenesis of ALS, the precise role of hypoxia in disease progression remains unknown. In this study, we detected higher expression levels of Hypoxia-inducible factor 1-alpha (HIF-1α), a key regulator of cellular responses to hypoxia, in the spinal cord of ALS patients and in the transgenic mice overexpressing the familial ALS-associated G93A SOD1 mutation (mSOD1^G93A^ mice) compared to controls. Single subcutaneous administration of sustained-release prostacyclin analog ONO-1301-MS to mSOD1^G93A^ mice abrogated the expression of HIF-1α in their spinal cords, as well as erythropoietin (EPO) and vascular endothelial growth factor (VEGF), both of which are downstream to HIF-1α. Furthermore, ONO-1301-MS increased the level of mature brain-derived neurotrophic factor (BDNF) and ATP production in the spinal cords of mSOD1^G93A^ mice. At late disease stages, the motor function and the survival of motor neurons of ONO-1301-MS-treated mSOD1^G93A^ mice was significantly improved compared to vehicle-treated mSOD1^G93A^ mice. Our data suggest that vasodilator therapy modulating local blood flow in the spinal cord has beneficial effects against ALS disease progression.

## Introduction

Amyotrophic lateral sclerosis (ALS) is a devastating disease characterized by progressive degeneration of motor neurons in the brain and spinal cord, resulting in muscle weakness. Although the precise mechanism of ALS remains unknown, it has become increasingly clear that ischemia and hypoxia are intimately involved in the pathogenesis of ALS^1–3^. In fact, Hypoxia-inducible factor 1-alpha (HIF-1α), a key regulator of cellular response to hypoxia, was highly expressed in the spinal cord of autopsy samples from ALS patients^4^. In addition, HIF-1α has been shown to play a critical role in the pathogenesis of animal models of ALS^4,5^.

Hypoxia is a risk factor for neurodegenerative diseases including Alzheimer’s disease^6^, and it is a stress which is closely related to the clinical course of ALS; defects in hypoxic signaling have been reported in ALS patients and mSOD1^G93A^ mice^7–10^. Respiratory status is one of the prognostic factors for ALS patients, and respiratory impairment is an early sign of disease onset in mSOD1^G93A^ mice^11,12^. Additionally, it has been becoming more and more clear that vascular changes are deeply involved in the pathogenesis of ALS^13–16^. Importantly, the blood-spinal cord barrier (BSCB), which is composed of blood vessels and central nervous system (CNS), is damaged in human ALS patients and mSOD1^G93A^ mice, and the findings in mice show that BSCB breakdown plays a role in early-stage disease pathogenesis. In fact, reduced microcirculation in the spinal cord of mSOD1^G93A^ mice at both early^13^ and late^16^ disease stage are reported. However, whether anti-hypoxia therapy, which increases the blood flow and oxygen supply to the motor neurons in the spinal cord, has a positive impact on neurodegeneration in ALS remains elusive.

ONO-1301 is a synthetic prostacyclin agonist that includes a five-membered ring and allylic alcohol but lacks typical prostanoid structures^17^. It has thromboxane-synthase inhibitory activity, leading to stronger and longer-lasting blood vessel dilatation *in vivo* than other endothelin receptor antagonists or phosphodiesterase-5 inhibitors. Previous studies have shown that ONO-1301 treatment, by decreasing vascular tone and increasing blood flow, ameliorates not only monocrotaline-induced pulmonary hypertension but also neurological dysfunction in rats with cerebral infarction^17,18^. More recently, a novel sustained-release ONO-1301 (ONO-1301-MS) has been developed by polymerizing ONO-1301 with poly (D,L-lactic-co-glycolic acid) (PLGA) microspheres^19^. A single subcutaneous injection of ONO-1301-MS resulted in sustained elevation of ONO-1301 levels for 12 weeks^20^.

In this study, we confirmed enhanced immunoreactivity of HIF-1α in the spinal cord of ALS patients and in transgenic mice overexpressing the familial ALS-associated G93A SOD1 mutation (mSOD1^G93A^ mice). To investigate the potential efficacy of anti-hypoxia therapy in ALS, we assessed the effect of ONO-1301-MS, a sustained-release form of ONO-1301, on disease progression in mSOD1^G93A^ mice.

## Results

### Elevated expression of HIF-1α suggests hypoxia in the spinal cords of ALS patients and transgenic ALS mice

We investigated HIF-1α expression as a marker for hypoxia and ischemia in autopsied spinal cord specimens from ALS patients and spinal cord samples of mSOD1^G93A^ mice. Increased HIF-1α immunoreactivity was detected in the anterior horn cells from ALS cases compared to controls (Supplementary Fig. S1a,b). Quantitative analysis showed that HIF-1α expression was significantly higher in the ALS group than in the control group (Supplementary Fig. S1c, P < 0.05, Mann-Whitney U test). Similar findings were detected in mSOD1^G93A^ mice compared to age-matched, wild-type mice (Supplementary Fig. S1d–f). These data are consistent with previous reports of hypoxia in the spinal cords of ALS patients^1^ and in the ALS mice model^4^.

### ONO-1301 protects against neurodegeneration in the mSOD1^G93A^ mouse model of ALS by suppressing hypoxic stress

**Figure 1:**
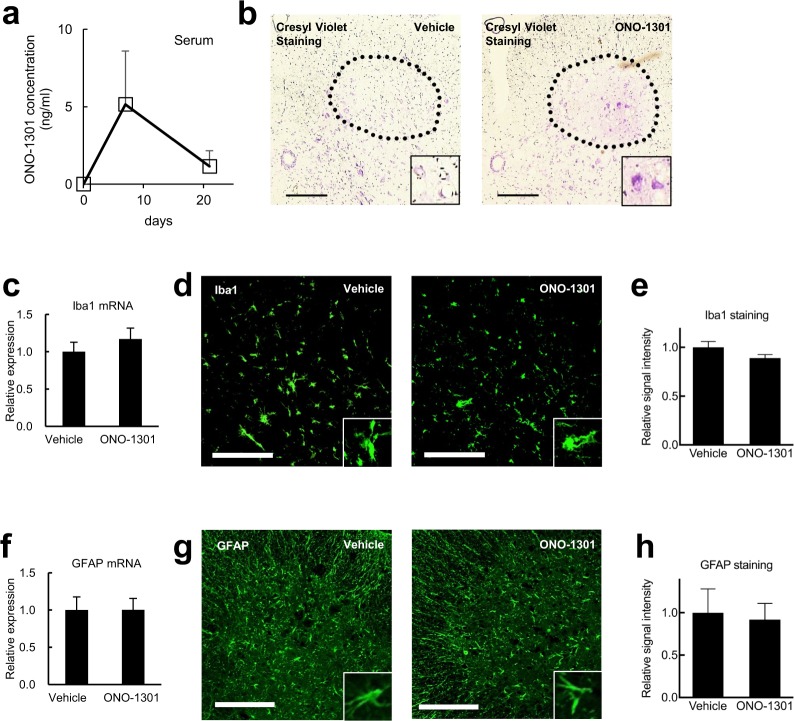
ONO-1301 preserved motor neurons in mSOD1^G93A^ mouse model of ALS without affecting neuroinflammation. (**a**) Serum concentrations of ONO-1301 after subcutaneous administration of ONO-1301-MS were evaluated. Effective blood concentrations were maintained after 21 days of administration (serum ONO-1301 concentration, 5.15 ± 3.45 ng/ml (7 days), 1.15 ± 1.02 ng/ml (21 days), mean ± SEM). (**b**) Histological analysis revealed that ONO-1301-MS administration preserved the number of motor neurons in the ventral horn of the lumbar spinal cords of mSOD1^G93A^ mice at 120 days of age. Representative images of sections that were stained with cresyl violet are shown. (**c**–**h**) Microgliosis and astrogliosis in the spinal cords of ONO-1301-MS–treated mSOD1^G93A^ mice and vehicle-treated control mice. Quantitative RT-PCR analyses for the expression of Iba1 (**c**) and GFAP (**f**) mRNAs in whole spinal cord samples (n = 5 mice per condition) from ONO-1301-MS–treated mSOD1^G93A^ mice and vehicle-treated control mice at 120 days of age. Sections of the lumbar cord were stained with an anti-Iba1 (**d**) or with an anti-GFAP antibody (**g**), and relative fluorescence signal intensities were quantified by ImageJ (**e**,**h**). Scale bar = 200 μm. Data are expressed as the mean ± SEM. *P < 0.05, **P < 0.01.

To explore the therapeutic effect of increased blood flow in ALS, we subcutaneously administered ONO-1301-MS, a sustained-release form of ONO-1301, or vehicle (PLGA) to mSOD1^G93A^ mice on day 64 of age. We were able to detect measurable concentrations of ONO-1301 in serum of ONO-1301-MS-treated mSOD1^G93A^ mice (Fig. 1a). Next, we measured the motor performance of both groups using the rotarod machine. ONO-1301-MS administration significantly improved the motor function of mSOD1^G93A^ mice at 17 weeks of age (*P* = 0.035, Mann-Whitney U test) and 20 weeks of age (*P* = 0.025, Mann-Whitney U test) (Supplementary Fig. S2a). Moreover, we found that ONO-1301-MS-treated mSOD1^G93A^ mice had increased body weight compared to control mice (Supplementary Fig. S2b, P < 0.01, repeated measures ANOVA for week 17 to week 20). Consistent with these observations, Nissl staining revealed that treatment with ONO-1301-MS resulted in increased motor neuron survival in the spinal cords of mSOD1^G93A^ mice at 17 weeks of age (Figs 1b and S2c). ONO-1301 had no significant impact on average survival time (Supplementary Fig. S2d, P = 0.99, log-rank test) and microglial/astroglial activation (Fig. 1c–h) in mSOD1^G93A^ mice. Collectively, these results suggest that ONO-1301 treatment improved motor function and ameliorated neurodegeneration of mSOD1^G93A^ mice at late stage of disease by suppressing hypoxic stress, though it did not affect neuroinflammation.

### ONO-1301 mitigated hypoxia in the spinal cord of mSOD1^G93A^ mice

**Figure 2:**
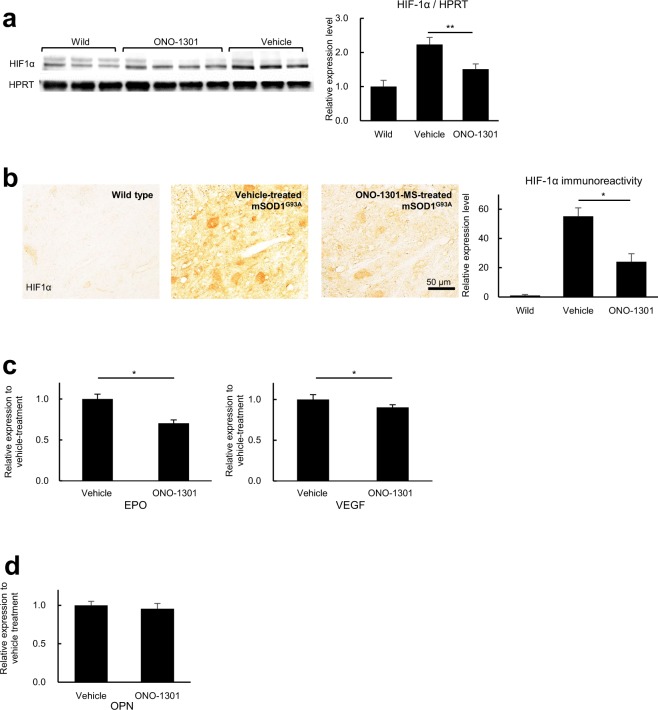
ONO-1301 ameliorates hypoxia in the spinal cord of mSOD1^G93A^ mice. (**a**) Western blot analysis revealed significantly decreased HIF-1α expression in the spinal cords of 120-day-old ONO-1301-MS–treated mSOD1^G93A^ mice relative to vehicle-treated mSOD1^G93A^ mice. (**b**) Fixed frozen sections of lumbar cord of mSOD1^G93A^ mice were stained with anti-HIF-1α antibodies. Representative images show reduced immunoreactivity of HIF-1α in 120-day-old ONO-1301-MS–treated mSOD1^G93A^ mice compared to age-matched vehicle-treated control mice (n = 3 mice per group). Scale bar = 50 μm. (**c**,**d**) Quantitative RT-PCR analyses for erythropoietin (EPO), vascular endothelial growth factor (VEGF) (c) and osteopontin (OPN) (**d**) in spinal cords of ONO-1301-MS–treated mSOD1^G93A^ mice and vehicle-treated control mice at 120 days of age (n = 5 per group). The expression levels of EPO (*P* = 0.014) and VEGF (*P* = 0.036) were significantly reduced in the ONO-1301-MS–treated group. Data are expressed as the mean ± SEM. *P < 0.05, **P < 0.01.

In order to confirm that the favorable effects by ONO-1301 were mediated through its anti-hypoxic activity, we examined the expression level of HIF-1α in the spinal cords of ONO-1301-MS-treated mSOD1^G93A^ mice and vehicle-treated controls. Western blot analysis of spinal cord extracts revealed significantly lower expression of HIF-1α following ONO-1301 treatment, suggesting that it alleviated hypoxia in the spinal cord of mSOD1^G93A^ mice (Fig. 2a). Next, we performed immunohistochemical staining to determine the localization of HIF-1α in the spinal cord of mSOD1^G93A^ mice. We observed the significantly decreased immunoreactivity against HIF-1α in the spinal cord of ONO-1301-MS-treated mSOD1^G93A^ mice (Fig. 2b). HIF-1α was largely localized in the cytoplasm of neuron-like cells. In addition, qPCR analysis demonstrated that ONO-1301 abrogated the transcription of erythropoietin (EPO) and vascular endothelial growth factor (VEGF), both of which are downstream molecules to HIF-1α, in the spinal cord of mSOD1^G93A^ mice (Fig. 2c), but not osteopontin (OPN), which is another marker of hypoxia^21^ (Fig. 2d). Collectively, these data suggest that ONO-1301 successfully ameliorated hypoxia in the spinal cord of mSDO1^G93A^ mice and alleviated neurodegeneration in this animal model of ALS.

### ONO-1301 increases ATP production, suggesting that it alleviates hypoxia in the spinal cord of mSOD1^G93A^ mice

**Figure 3:**
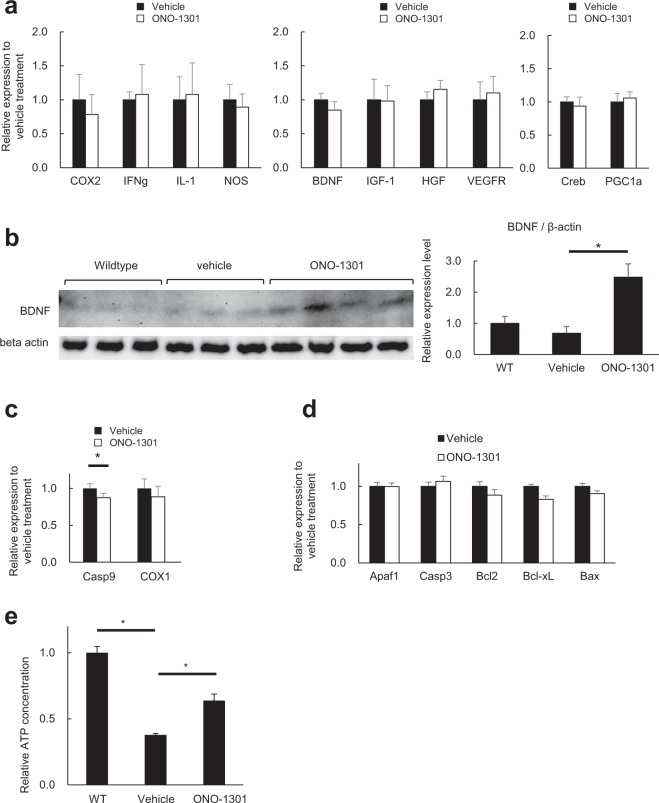
ONO-1301 increased mature BDNF and ATP production in the spinal cord of mSOD1^G93A^ mice. (**a**) Relative mRNA expression levels of cyclooxygenase 2 (COX2), interferon gamma (IFN-g), interleukin 1 beta (IL-1), nitric oxide synthase 2 (NOS), brain-derived neurotrophic factor (BDNF), insulin-like growth factor 1 (IGF-1), hepatocyte growth factor (HGF), vascular endothelial growth factor receptor (VEGFR), cAMP response element binding protein (Creb), and peroxisome proliferator-activated receptor gamma coactivator 1-alpha (PGC1a) in the spinal cord of 120-day-old ONO-1301-MS–treated mSOD1^G93A^ mice and vehicle-treated controls. (**b**) Western blot analysis revealed significantly increased level of mature BDNF in the spinal cords of 120-day-old ONO-1301-MS–treated mSOD1^G93A^ mice relative to vehicle-treated mSOD1^G93A^ mice. (**c**) Relative mRNA expression levels of caspase-9 (Casp9) and cyclooxygenase 1 (COX1) in the spinal cord of 120-day-old ONO-1301-MS–treated mSOD1^G93A^ mice and vehicle-treated controls. (**d**) Relative mRNA expression levels of Apoptotic protease activating factor 1 (Apaf1), caspase 3 (Casp3), B-cell lymphoma 2 (Bcl2), B-cell lymphoma-extra large (Bcl-xL), and BCL2 associated X (Bax) in the spinal cord of 120-day-old ONO-1301-MS–treated mSOD1^G93A^ mice and vehicle-treated controls. (**e**) ATP concentrations in spinal cords of ONO-1301-MS–treated mSOD1^G93A^ mice and vehicle-treated mSOD1^G93A^ mice at 12 weeks of age (n = 3 mice per group) after 3 weeks of treatment. The values were normalized to basal levels in wild-type mice or in vehicle-treated mSOD1^G93A^ mice. Data are expressed as the mean ± SEM. *P < 0.05.

Following cerebral ischemia, various molecules related to inflammation, apoptosis, trophic factors, and transcription factors are induced, and some of these molecules are known to be involved in neurodegeneration of ALS. To clarify the mechanisms of actions related to the efficacy of ONO-1301, we compared the expression levels of molecules associated with these pathways in the spinal cord of ONO-1301-MS-treated and vehicle-treated mSOD1^G93A^ mice using semi-quantitative RT-PCR. ONO-1301 had no significant effects on the expression levels of molecules related to inflammation, trophic factors, and transcription factors involving ischemia such as cAMP response element binding protein (Creb) and proliferator-activated receptor gamma coactivator 1-alpha (PGC1a) (Fig. 3a). Importantly, ONO-1301 administration significantly increased the level of mature BDNF in the spinal cord of mSOD1^G93A^ mice (Fig. 3b). Although ONO-1301 significantly decreased the expression level of caspase-9 (Casp9), a key molecule in the mitochondrial apoptosis pathway (Fig. 3c), western blot analysis did not reveal any significant effects on the cleavage of active caspase-9 (data not shown). Similarly, ONO-1301 had no obvious effect on the transcriptional levels of other molecules that are involved in apoptosis (Fig. 3d).

Given that ischemia and hypoxia usually cause deprivation of mitochondrial ATP production, we sought to determine whether ONO-1301 treatment increased ATP concentration in the spinal cord of mSOD1^G93A^ mice. We confirmed that ONO-1301-MS treatment significantly restored ATP concentration, suggesting increased ATP production from the mitochondria and improved blood supply in the spinal cord of mSOD1^G93A^ mice (Fig. 3e). Taken together, these data suggest that ONO-1301 alleviated hypoxia in the spinal cords and prevented neurodegeneration in mSOD1^G93A^ mice partly through increasing BDNF and restoring mitochondrial functions.

## Discussion

In this study, we showed that treatment with a prostacyclin agonist successfully ameliorated the motor functions of mSOD1^G93A^ mice and prevented their body weight loss. The beneficial effects of prostacyclin agonist treatment in ALS mice were partly mediated by alleviating hypoxia and hypo-perfusion in the spinal cord, leading to the increased ATP production. Moreover, we have shown that increasing blood flow in hypoxic regions of the spinal cord might play an important role in protecting against neurodegeneration in an animal model of ALS. To the best of our knowledge, this is the first report that suggests anti-hypoxia treatment could be a therapeutic option for the treatment of ALS.

We showed that HIF-1α expression was decreased in the spinal cord of ONO-1301-MS-treated mSOD1^G93A^ mice. This was accompanied by lower transcription levels of EPO and VEGF, which have been reported to be neuroprotective in the pathogenesis of ALS^22^. Anti-hypoxia therapy using ONO-1301, which improved mitochondrial energy production, could counteract the negative effects of downregulating neuroprotective factors including VEGF. In fact, decrease in transcriptional level of VEGF by ONO-1301 was subtle.

An increasing number of researchers have revealed that hypoxia and ischemia are intimately involved in the pathogenesis of ALS^16^. Recently, Sun *et al*. reported that repeated transient ischemia upregulates TAR DNA-binding protein 43 (TDP-43), which is a key molecule in the pathogenesis of ALS^23^. Another group reported that overexpression of TDP-43 is toxic to motor neurons^24^. Collectively, these reports suggest that anti-hypoxia therapy using ONO-1301 could ameliorate the motor symptom of ALS by reducing the expression levels of TDP-43. In addition, overexpression of mutant SOD1^G93A^ in human neuronal cells decreased cell viability and disabled autophagy when cultured in a low O_2_ environment^9^. Given that motor neurons in patients with ALS have a higher susceptibility to hypoxic stress, anti-hypoxia treatment could be a useful target for the treatment of ALS.

Regarding the mechanisms by which ONO-1301 had a positive impact on neurodegeneration in animal models of ALS, we consider that not only its anti-hypoxia effect but also its effect against pericytes is important. In fact, several reports suggest that pericytes are deeply involved not only in the physiological activity of brain but also in the pathogenesis of neurodegenerative diseases such as ALS^25–29^. Notably, pericytes extend the survival of ALS mice^27^, suggesting that increasing the number of pericytes could be a therapeutic option in the treatment of ALS. Importantly, prostacyclin recruits pericytes^30^ and suppress the loss of pericytes in central nervous system^31^. Collectively, these results raise the possibility that ONO-1301 suppressed neurodegeneration in mSOD1^G93A^ mice partly through acting on pericytes.

ONO-1301-MS treatment successfully prevented the loss of motor neurons in the spinal cord of mSOD1^G93A^ mice at end-stage but failed to prolong their overall survival. This may be partly due to the inability of ONO-1301 to fully alleviate the hypoxic stress induced in mSOD1^G93A^ mice. Indeed, ONO-1301 did not demonstrate significant suppression of pro-inflammatory (Fig. 3a) nor pro-apoptotic molecules (Fig. 3d), which are strongly implicated in the pathogenesis of ALS^32–36^. More potent anti-hypoxic therapies or combination therapies targeting cascades other than hypoxia may be required to prolong overall survival.

We showed that ONO-1301-MS treatment increased the blood flow and suppressed hypoxia in the spinal cord of mSOD1^G93A^ mice. On the other hand, it might have increased the leakage of neurotoxins by increasing blood flow in compromised microcapillaries in the spinal cord of mSOD1^G93A^ mice, leading to the minimal neuroprotective effect. In fact, recent reports by Winkler *et al*. and Zhong *et al*. suggest that blood-spinal-cord barrier (BSCB) in mSOD1^G93A^ mice is compromised at early-stage of the disease, and activated protein C (APC) and its analog restored the disrupted BSCB in mSOD1^G93A^ mice and contributed to protecting from motor neuronal degeneration^14,15,28^. Based on their results and ours, combination therapy with ONO-1301-MS and APC could be a more promising option for the treatment of ALS by increasing blood flow in repaired microcapillaries in the spinal cord.

Although additional studies are needed to clarify the potential role of hypoxia in ALS, our findings highlight the therapeutic potential of targeting the hypoxia/hypo-perfusion axis for the treatment of ALS. Thus, a treatment modality that inhibits hypoxia holds great therapeutic promise for treating ALS patients.

## Methods

### Tissue sources of autopsied spinal cord

The research presented in this study was approved by the Ethics Committee of Osaka University Graduate School of Medicine (permit number 10038, 25-079-005, Biken-AP-H21-28-0). Lumbar cord specimens were obtained at autopsy from ALS patients and other neurological disease control patients (one multiple system atrophy (MSA) and two familial amyloid polyneuropathy (FAP) patients). Consent for autopsy was obtained from legal representatives for all subjects in accordance with the local institutional review board requirements, which were approved by the Ethics Committee of Osaka University Graduate School of Medicine. All neuropathologic analyses were performed by trained neuropathologists.

### Preparation of ONO-1301-MS

ONO-1301 was synthesized by ONO Pharmaceutical, (Osaka, Japan) as previously described^37–39^. A sustained-release form of ONO-1301 (ONO-1301-MS) was generated by encapsulating ONO-1301 with poly (D,L-lactic-co-glycolic acid) (PLGA) as described previously^19,40^. Briefly, polyvinyl alcohol aqueous solution (poor solvent of PLGA) was put into a glass vessel. An ethanol/dichloromethane solution containing PLGA and ONO-1301 was dropped into the poor solvent while stirring to form an oil-in-water emulsion. Dichloromethane was then evaporated off by stirring at room temperature. After centrifugation and washing, ONO-1301-PLGA microspheres were isolated by lyophilization.

### Animals and treatment with ONO-1301-MS

mSOD1^G93A^ mice were obtained from The Jackson Laboratory (strain designated: B6SJL-TgN(SOD1-G93A)1Gurd/J) and backcrossed with C57BL/6 mice for at least ten generations. ONO-1301-MS (250 mg/kg) was administered via subcutaneous injections into the backs of mice as previously described^39^. For the measurement of ATP, ONO-1301-MS was administered to 9-week-old mSOD1^G93A^ mice and they were sacrificed after 3 weeks of administration. For the other experiments, ONO-1301-MS was administered at 64 days of age.

### Assay of serum level of ONO-1301

The whole blood was taken from mice and allowed to clot by leaving it undisturbed at room temperature for 30 minutes. The clot was removed by centrifugation at 1000 *g* for 10 min in a refrigerated centrifuge. Serum ONO-1301 levels were measured by liquid chromatography with tandem mass spectrometric assay.

### Motor neuron counts

mSOD1^G93A^ mice at 120 days were sacrificed and perfused with 4% paraformaldehyde in PBS, and lumbar spinal cords were dissected out. Spinal cords were dehydrated in increasing alcohol concentrations, before embedding in paraffin. For motor neuron counts, 20 serial horizontal sections of the L5 segment of spinal cord (10 μm thick) were cut using a microtome and mounted onto slides. Cresyl violet staining was performed on the 5th, 10th, 15th and 20th serial sections and motor neurons in both ventral horns on each section were counted within an area defined by a horizontal line through the central canal. Four male animals in ONO-1301-MS-treated group and 6 male animals in vehicle-treated group were analyzed. These experiments were conducted in a blinded fashion.

### Immunohistochemistry

Fixed frozen sections of spinal cord from mSOD1^G93A^ mice (10 μm thick) were prepared as previously described^32^. Hypoxia-induced factor 1-alpha (HIF-1α) expression was evaluated in paraffin-embedded 6-μm–thick sections of the lumbar cord from ALS patients and 10 μm–thick fixed frozen sections of spinal cord of mSOD1^G93A^ mice as previously described^41^. The following primary antibodies were used: rabbit anti–HIF-1α polyclonal antibody (1:100; Novus Biologicals), rabbit anti–Iba1 polyclonal antibody (1:1000, Wako) and Alexa Fluor 488®–conjugated mouse anti–glial fibrillary acidic protein (GFAP) monoclonal antibody (1:200; Cell Signaling Technology). The following secondary antibodies were used: AlexaFlour488-conjugated goat anti–rabbit IgG antibody (1:500, Invitrogen) and goat anti–rabbit immunoglobulins conjugated to peroxidase-labeled dextran polymer (Dako Envision+, Dako). For HIF-1α staining, reaction products were visualized with 3,3′-diaminobenzidine tetrahydrochloride (Vector Laboratories). The relative optical densities of HIF-1α immunoreactivity were quantified using ImageJ. The fluorescently labeled sections were examined using an LSM 510 confocal microscope (Zeiss).

### Western blotting

Western blot analysis was performed as previously described^42^. Samples were lysed with NP-40 buffer [PBS, 01% TritonX, 05% sodium deoxycholate, 01% sodium dodecyl sulfate (SDS), 50 mM Trix-HCl and 150 mM NaCl, pH 80] containing protease inhibitors (20 mg/ml aprotinin and 1 mM phenylmethylsulfonyl fluoride), 2-mercaptoethanol and 1 mM sodium orthovanadate. Equal concentrations of protein were resolved on 10% SDS-polyacrylamide gels, and then transferred onto Poly-vinylidene Difluoride (PVDF) membranes (ATTO, Tokyo, Japan). The blots were incubated at 4 °C overnight with one of the following primary antibodies: rabbit anti-HIF-1α polyclonal antibody (1:500; Novus Biologicals), rabbit anti–HPRT monoclonal antibody (1:2000; abcam), rabbit anti-BDNF monoclonal antibody (1:500; abcam), mouse anti-β-actin monoclonal antibody (1:10000; Sigma-Aldrich). The blots were subsequently incubated with the appropriate horseradish peroxidase–conjugated secondary antibodies for 90 min and visualized using SuperSignal West Femto Maximum Sensitivity Substrate (Thermo Fisher Scientific, Waltham, MA, USA). The image of each band was captured and analyzed using Image Gauge (Fuji Film, Japan) and ImageJ.

### RNA extraction and RT-qPCR analysis

Total RNA and cDNA from spinal cords of ONO-1301-MS-treated mSOD1^G93A^ mice at 120 days of age and age-matched control mice were generated as previously described^35^. cDNA was amplified using SYBR Premix Ex Taq II (Takara Bio) and analyzed as previously described^36^.

### Measurement of ATP

Spinal cord tissue was homogenized in sterilized ultrapure water on ice and the lysates were centrifuged at 1000 *g* for 5 min. Extractions of ATP were performed using an AMERIC-ATP kit (AMERIC, Japan), and the fluorescence in each sample was measured using Lumat3 (LB9508, BERTHOLD).

### Statistical analysis

Student’s *t*-test was used to compare two groups and an ANOVA was used to compare more than two groups. Data are expressed as the mean ± SEM. *P*-values less than or equal to 0.05 were considered statistically significant.

### Ethics approval and consent to participate

The present study was conducted according to the revised Declaration of Helsinki and Good Clinical Practice guidelines and was approved by the local ethics committees. Informed consent was given by all study participants or their legal representative. All animal experiments were performed in compliance with the Japanese national guidelines and the guidelines of Osaka University, which specifically approved this study (Permit number: Biken-AP-H21-28-0).

## Supplementary information

Supplementary Figures

## Data Availability

The datasets used and/or analyzed during the current study are available from the corresponding author on reasonable request.
