# Supplementary material for: Single injection of sustained-release prostacyclin analog ONO-1301-MS ameliorates hypoxic toxicity in the murine model of amyotrophic lateral sclerosis
Source: Sci Rep. 2019 Mar 27;9:5252. doi: 10.1038/s41598-019-41771-4 (PMC6437213; doi:10.1038/s41598-019-41771-4)
Supplement: Supplementary file 1 — Supplementary Figures [file 41598_2019_41771_MOESM1_ESM.pdf]

Supplementary Information (Supplementary Figures)

Title:
Single injection of sustained-release prostacyclin analog ONO-1301-MS ameliorates hypoxic toxicity in the murine model of amyotrophic lateral sclerosis

Author list:
Satoru Tada[1,2,#], Tatsusada Okuno[1,#,*], Mikito Shimizu[1,#], Yoshiki Sakai[3], Hisae Sumi[1], Makoto Kinoshita[1], Kazuya Yamashita[1], Eri Sanda[1], Chi-Jing Choong[1], Akiko Namba[1], Tsutomu Sasaki[1], Toru Koda[1], Kazushiro Takata[1], Shigeru Miyagawa[3], Yoshiki Sawa[3], Yuji Nakatsuji[1], Hideki Mochizuki[1]

Institutional addresses:
1 Department of Neurology, Osaka University Graduate School of Medicine, 2-2, Yamadaoka, Suita, Osaka 565-0871, Japan
2 Department of Neurology, Tanaka Naika Medical Clinic, 2-7-28, Matsunohama-cho, Izumiotsu, Osaka, 595-0072, Japan
3 Department of Cardiovascular Surgery, Osaka University Graduate School of Medicine, 2-2, Yamadaoka, Suita, Osaka 565-0871, Japan

*Correspondence should be addressed to Dr. Tatsusada Okuno,

Department of Neurology, Graduate School of Medicine, Osaka University, 2-2 Yamada-oka, Suita, Osaka 565-0871, Japan
E-mail: okuno@neurol.med.osaka-u.ac.jp

#These authors equally contributed to this work.

Supplementary Figure-S1

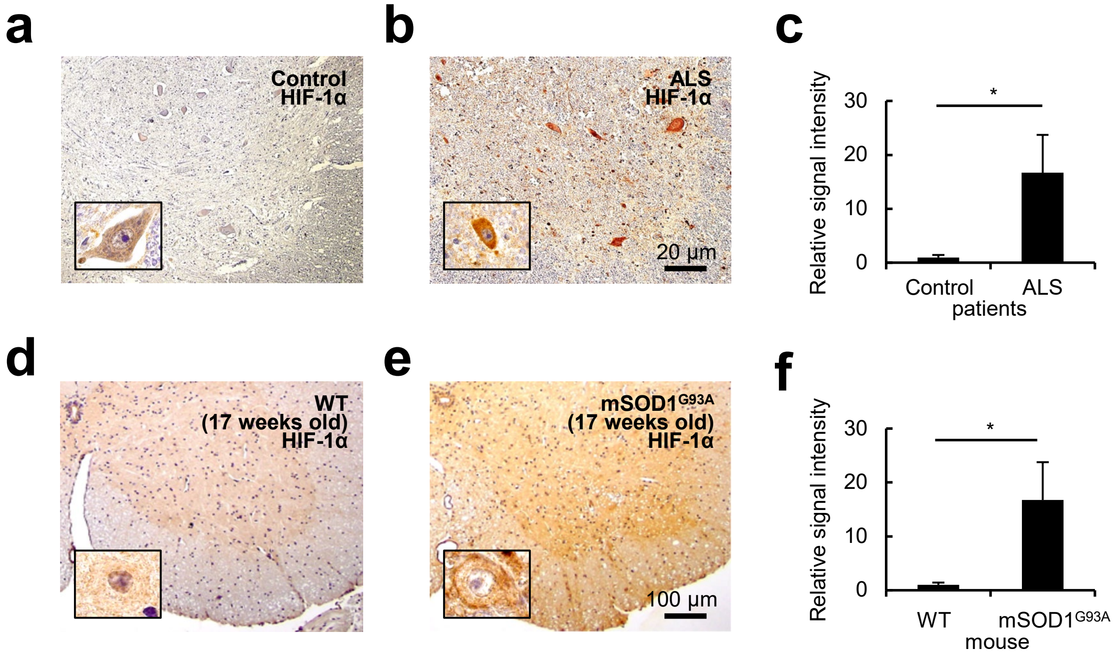

Supplementary Figure S1
Elevated expression of HIF-1α in the spinal cords of ALS patients and transgenic ALS mice.
(a, b) Representative images of HIF-1α expression in lumbar cords from control patients diagnosed multiple system atrophy (MSA) (a) and ALS patients (b). Hematoxylin was used to counterstain the cell nuclei. Scale bar = 20 µm. (c) Quantification of HIF-1α immunoreactivity in three sporadic ALS patients who did not have a history of respirator use (median age at death 62 years [range, 60–65 years], postmortem interval 4 h [2–8 h], disease duration 2.5 years [1–5 years]) and three control patients (one MSA and 2 familial amyloid polyneuropathy patients (median age at death: 55 years [47–64 years], postmortem interval: 7 h [2–17 h], disease duration: 4.5years [4–6 years]). (d, e) Representative images showing HIF-1α expression in lumbar spinal cords from wild-type littermates (d) and mSOD1^{G93A} mice (e) at 115 days of age. Scale bar = 100 µm. (f) Quantification of HIF-1α expression in the lumbar spinal cord of mSOD1^{G93A} mice. Data are expressed as the mean ± SEM. *$P < 0.05$.

Supplementary Figure-S2

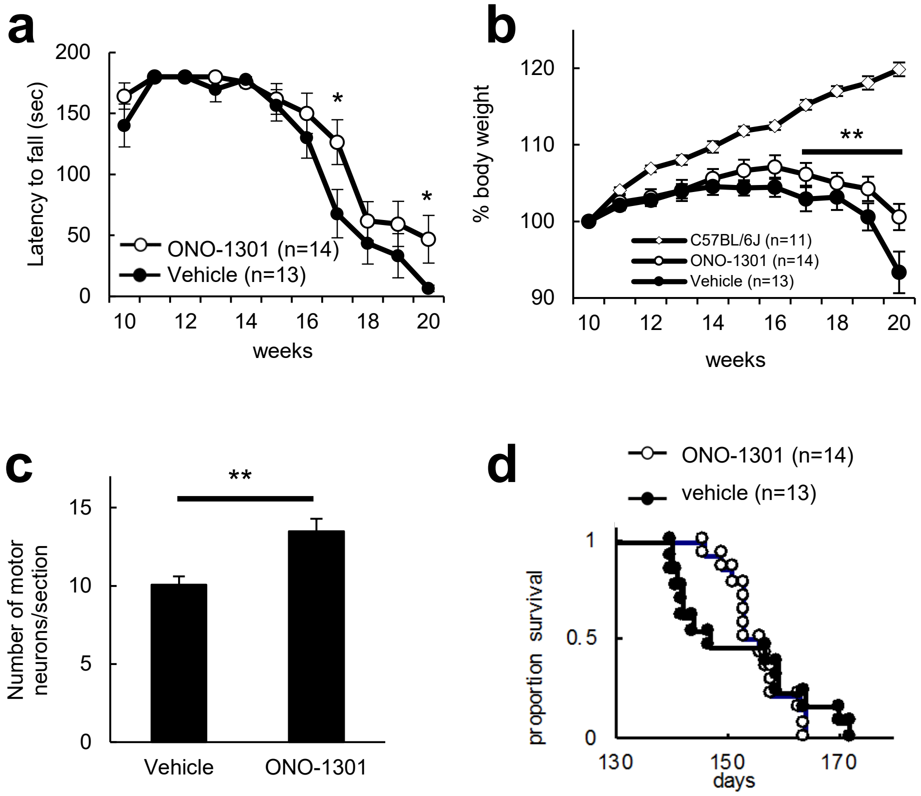

Supplementary Figure S2

ONO-1301 protects against neurodegeneration in mSOD1^{G93A} mice.

(a) Motor performance of ONO-1301-MS-treated mSOD1^{G93A} mice is shown by open circles (n = 14, 6 male mice and 8 female mice), and that of vehicle-treated mSOD1^{G93A} mice is shown by filled circles (n = 13, 6 male mice and 7 female mice). ONO-1301-MS–treated mSOD1^{G93A} mice displayed significantly improved motor performance at 17 and 20 weeks of age. (b) Changes in mean body weight of C57BL/6J mice (n = 11), ONO-1301-MS–treated mSOD1^{G93A} mice (n = 14) and vehicle-treated mSOD1^{G93A} mice (n = 13) are shown. (c) Histological analysis revealed that ONO-1301-MS administration significantly preserved motor neurons in the ventral horn of the lumbar spinal cords of mSOD1^{G93A} mice at 120 days of age (n = 4 for ONO-1301-MS-treated animals and n = 6 for vehicle-treated controls, $P < 0.01$). (d) Kaplan-Meier survival curve. ONO-1301-MS treatment did not affect the survival of mSOD1^{G93A} mice (average survival time: 155.57 ± 1.47 days in ONO-1301-MS-treated group, n = 14; 152.08 ± 3.29 days in vehicle-treated group, n = 13) ($P = 0.99$). Data are expressed as the mean ± SEM. *$P < 0.05$, **$P < 0.01$.
